# Vienna Letter

**Published:** 1882-08

**Authors:** W. T. Belfield

**Affiliations:** Vienna


ARTICLE X.

VIENNA, JULY 3, 1882.

EDITORS MEDICAL JOURNAL AND EXAMINER:

The tubercle bacillus is still the lion of the day, and bacillus hunting the chief occupation of all interested in pathology. That the organisms are present, both in tuberculous tissues and tuberculous sputum, is universally acknowledged. Hitherto no one has repeated, so far as I can ascertain, Koch's experiments in cultivation and inoculation; and upon these rests of course, the asserted causal relation of the bacteria to the disease. Yet it is highly probable that in many German laboratories these experiments, necessarily slow, are being conducted. Indeed, Koch's previous work has been so thorough and unassailable, his conclusions so cautious and reserved, that no one is inclined to doubt his veracity or his judgment in the present instance. In a recent meeting of the Royal Society of Physicians here, Billroth proclaimed his acceptance of Koch's conclusions, observing, at the same time, that since the experiments of Villemin and others, he has been expecting the discovery of a vegetable parasite as the cause of the disease—since its infectious nature was by these experiments clearly proven.

Several vexed questions in pathology must now find an early solution. First, it must be already regarded as proven, that tuberculosis and consumption are, etiologically at least, identical. Niemeyer's famous remark—" The greatest danger for consumptives is that they may become tuberculous," may retain some clinical, but no pathological value. Again, we may perhaps now be able to prove, by the discovery of the bacilli in scrofulous glands, the already highly probable identity of "scrofula" with tuberculosis. Lupus vulgaris, moreover, is by many ascribed to the scrofulous diathesis—indeed, I was told a few days ago that a Berlin pathologist has already found the bacilli in lupus nodules. We shall see. Clinically, Koch's discovery does not as yet promise much, except in the way of prophylaxis; that a child with hereditary tendency to consumption should avoid intercourse with individuals already consumptive, has been long known (though but little practiced); now, however, we know that infection can occur through the mother's milk; perhaps even from handkerchiefs and other articles used by a consumptive—and should act accordingly. Whether the earliest stage of the disease can be recognized by the microscope sooner than by the stethoscope, has not as yet been determined, though it seems highly probable, when one sees the myriads of bacilli in every drop of sputum from a patient who presents but limited areas of pulmonary degeneration. By the way, the detection of bacilli in sputum is much easier than in tissue; moreover, the experience of microscopists here, with which my own observation coincides, is that the organisms are more often found in cheesy degeneration than in the fresh miliary tubercle with its giant-cells. Koch, however, usually finds them in the giant-cells.

For the detection of the tubercle bacilli, the best method up to date is Ehrlich's, which has doubtless long ago reached you. Yet, as it requires considerable skill, and at least forty-five minutes' time, it is hardly adapted to general clinical use. Baumgarten has recently proposed a method which has the advantage of brevity—ten minutes—but the disadvantage that the bacilli are all but invisible. By Ehrlich's method the bacilli are distinctly visible with 400 diameters, and without the abbé's illuminating apparatus, oil immersion, etc., which Koch's original method required. Indeed, Koch himself now uses Ehrlich's method.

In the recent surgical congress at Berlin, Langenbeck arrayed himself among the defenders of iodoform, warning, at the same time, against the use of more than ten or fifteen grams on one individual. An interesting discussion of the pylorus resection followed. Of eighteen patients operated on for cancer, three still live; one, a girl, whose pylorus was removed by Rydygier for cicatricial stricture after ulcer, was exhibited to the congress, in "blooming" health, as the report states. Billroth considered the operation in such cases justifiable, in opposition to Richter, who thought the time had not yet come for such a measure. Langenbeck had in one case removed the pancreas, with the adherent pylorus (result not stated, but easily imagined). Billroth expressed his surprise that so many cases had been already operated; thinks that but few cases (one in fifty or sixty) are fit for operation, because the diagnosis is made too late; has himself made twenty exploratory incisions, without operating—the exploration was in one case fatal. As to the technique, Billroth lays great stress upon an accurate adjustment of the edges of the mucous membrane, so that the intestinal and gastric contents shall not come in contact with the wound. He rejects catgut for sutures, because it stretches too easily; uses silk.

Sonnenburg exhibited a case of ectopia vesicæ which he had successfully operated by removing the bladder and sewing the ureter into the (open) urethra. Thiersch reported, in favor of his own method, that of twenty cases which he had operated in fifteen years, sixteen were healed; four died.

In Vienna, I have seen nothing of especial interest recently in the clinical way except two unpublished cases of Prof. Dittel's. In a case of very old and very obstinate cystitis, which had resisted all the usual therapeutic measures, Dittel made external urethrotomy in the membranous portion, introduced a catheter á demeure, and washed out with boracic acid solution. Within two months the patient went home with healthy urethra and bladder. In a litholopaxy last week Dittel introduced the No. 30 catheter for washing out the bladder. Five days later there was thrombosis of the dorsal vein of the penis, soon followed by chills, and by gangrene of the glans. Patient (man of sixty-four years) is expected to die in a few days of septicæmia. This is, I believe, the first "*malheur*" that Prof. Dittel has experienced in nearly a hundred lithotrities.

Billroth was recently invited to take Langenbeck's chair in Berlin. He promptly declined, and received, in consequence, an ovation from the Vienna students. First, a handsomely engrossed complimentary address was presented to him in the aula of the university, in the presence of numerous professors and an immense audience; the same evening followed a torchlight procession by the students, including about 800 torch-bearerers, with band, glee-club, ferocious-looking "Burschen" in high boots, foxtail caps, and carrying drawn swords. On this, as on every other occasion where there is anything to be seen, the populace was out in force, crowding the streets for hours before the procession appeared. After a serenade, and a few cordial words by Billroth, the torches disappeared, and the bearers thereof spent the rest of the night in discussing the event, Billroth, and Pilsner beer.

The writer had the pleasure of meeting Dr. Allen, of Rush college, some three weeks ago. He and Mrs. Allen seemed well pleased with our old Kaiserstadt.

W. T. BELFIELD.

